# The effect of aspartame and pH changes on the erosive potential of cola drinks in bovine enamel: An in vitro study

**DOI:** 10.4317/jced.54963

**Published:** 2018-09-01

**Authors:** Daniela Rios, Franciny-Querobim Ionta, Rafael Rebelato, Maisa-Camillo Jordão, Linda Wang, Ana-Carolina Magalhães, Heitor-Marques Honório

**Affiliations:** 1 Departments of Pediatric Dentistry, Orthodontics and Public Health; 2 Departments of Restorative Dentistry, Endodontics and Dental Materials; 3 Departments of Biological Sciences

## Abstract

**Background:**

The aim of this study was to clarify the mechanism behind the different erosive potential of regular and light cola drinks: pH difference and/or aspartame presence.

**Material and Methods:**

Sixty bovine enamel blocks were randomly divided into 5 groups: RC - regular cola, RCpH - addition of base to increase regular cola pH, RCAS - addition of aspartame to regular cola, LC - light cola, and LCpH - addition of acid to decrease light cola pH. Two-thirds of the blocks surface was coated with nail varnish for reference. The samples were daily subjected to four erosive challenges for 2 minutes. Between the erosive challenges (2h) and overnight the samples were maintained in artificial saliva. The response variable was the percentage surface hardness change (%SHC) after 1st experimental day and enamel surface loss (µm) measured at the 5th day by profilometry. Data were analyzed by ANOVA and Tukey’s test (*p*<0.05).

**Results:**

Independently of the cola modifications, all groups promoted similar hardness change of enamel surface. RC promoted higher enamel loss (6.69±0.71µm) than LC (4.80±0.77µm). The acid addition to light cola (LCpH: 6.60±1.78µm) significantly enhanced its erosive potential, which did not differ from RC. The base addition to regular cola (RCpH-4.00±0.64µm) resulted in similar wear to LC. The addition of aspartame to the regular cola (RCAS 5.44±0.65µm) resulted in similar wear to LC and RC.

**Conclusions:**

The data suggest that the pH alteration has a major impact on the erosive potential of cola drinks, however, the sweetener also has some influence.

** Key words:**Tooth erosion, dental enamel, soft drinks, ph, sweetener.

## Introduction

Nowadays, clinical evidences highlight tooth erosion as a relevant dental health imbalance. The prevalence of erosive wear varies widely among studies (1), however there are some evidences from longitudinal studies showing that its occurrence and severity has increased over the years (2-4).

 Dental erosion can be defined as a pathological dissolution of dental hard tissues due to their interaction with non-bacterial acids (5). It is important to highlight that the acid leads to the softening of the tooth surface, which becomes more susceptible to removal by abrasion and attrition (6). Erosive foods and soft drinks, can have numerous components with complex composition, representing an important risk factor for the development of erosive lesions (7,8). Soft drink consumption has been correlated with severity of dental erosion among adolescents (9,10). Since the worldwide consumption of soft drinks is continually increasing (11), its erosive potential, specially of the popular cola drinks, needs to be clarified.

Previous in situ studies showed that immersion of enamel blocks in light cola resulted in less wear when compared to its immersion in regular cola (12,13). It was speculated that such less aggressive erosive effect on enamel by light cola could be due to the higher pH value or the presence of inhibitors of erosion, which could be the aspartame (12,13). The knowledge of the chemical properties responsible for the less erosive potential of the light cola could generate insights to further modifications of erosive soft drinks. Therefore, this in vitro study was designed to test these hypotheses by the modification of the pH of cola drinks and by the addition of aspartame.

## Material and Methods

-Blocks preparation

Enamel blocks (4 X 4 X 3 mm) were prepared from the labial surfaces of bovine incisors crowns. The blocks were cut using a ISOMET low speed saw cutting machine (Buehler Ltd., Lake Bluff, IL, USA) with two diamond disks (Extec Corp., Enfield, CT, USA), which were separated by a 4-mm thickness spacer. The blocks’ surfaces were ground flat with water-cooled silicon carbide discs (320, 600, and 1200 grade papers; Buehler, Lake Bluff, IL, USA), and polished with felt paper wet by diamond spray (1 mm; Buehler, Ltd., Lake Bluff, IL, USA). The blocks were cleaned using an ultrasonic device for 2 min and checked regarding the presence of white spots and cracks using a microscope (x40).

-Surface hardness for selection

A surface Knoop hardness test was performed (5 indentations in different regions of the slab, 25 g, 5 s, HMV-2; Shimadzu Corporation, Tokyo, Japan) to select 60 bovine enamel blocks (SHi) with hardness values between 320 and 380 KgF/mm2 (mean surface hardness of 356 ± 20 KgF/mm2).

-Experimental Design

The factor under evaluation was cola drink modification in five levels (n=12): (RC) erosion with regular cola (pH 2.6), (RCpH) erosion with regular cola with pH alteration (addition of NaOH to get similar pH to light cola; pH 3.0), (RCAS) erosion with regular cola with the addition of aspartame (24mg/100mL; pH 2.6), (LC) erosion with light cola (pH 3.0) and (LCpH) erosion with light cola with pH alteration (addition of H3PO4 to get similar pH to regular cola; pH 2.6). The response variables were percent of surface hardness change (%SMH) after the 1st day, and depth of enamel surface loss, after the fifth day of erosive challenge. Sample size calculation was based on a pilot study, regarding enamel wear. A sample size of 12 enamel blocks per group was estimated based on an α-error of 5%, β-error of 20%, 0.68 μm estimated standard deviation, and 1 μm minimum detectable difference in means.

-Cola drinks modifications

The preparation for cola modifications was performed before each erosive challenge. For group RC-regular cola, a degassed bottle was used (Coca-Cola/pH 2.6; companhia Fluminense de Refrigerantes, Porto Real, RJ, Brazil) in each erosive challenge. In the RCpH group, 1 M of NaOH solution (Merck, Darmstadt, Germany) was added to 360 ml of degassed cola drink (Coca-Cola/pH 2.6) until the pH increased to 3.0. For RCAS group, 86.4 mg of aspartame (NutraSweet. Monsanto, São Paulo, SP, Brazil) was added to 360 ml of degassed cola drink (Coca-Cola/pH 2.6), in order to reproduce the same aspartame concentration of Light Cola. The addition of aspartame did not change the colas pH. A degassed bottle of light cola (Coca-Cola Light/pH 3.0; Companhia Fluminense de Refrigerantes, Porto Real, RJ, Brazil) was used in LC-light cola group. In the LCpH group 0.1 M of phosphoric acid solution (Merck, Darmstadt, Germany) was added to 360 ml of degassed light cola drink (Coca-Cola Light/pH 2.9) until the pH decreased to 2.6 (same pH of regular cola).

-Erosive cycling

Prior to the experiment, two layers of nail varnish were applied on two-thirds of the surface of each block to maintain reference surfaces for enamel loss determination after the experimental phase. The central third was left uncovered. Twelve blocks per group were subjected to a 5-day erosive cycling under room temperature (25ºC). Erosion was performed by immersion of the blocks in each studied cola drink (30 ml/sample, unstirred) four times daily for 2 min. The cola drinks were renewed (4 times per day) in each erosive attack. After demineralization, the blocks were rinsed with tap water and transferred into artificial saliva (30 ml/sample, unstirred, 25°C) for 120 min. After the last daily erosive challenge, the blocks were stored in artificial saliva overnight (14). The artificial saliva was renewed daily and consisted of 0.2mM glucose, 9.9mM NaCl, 1.5mM CaCl2.2H2O, 3mM NH4Cl, 17mM KCl, 2mM NaSCN, 2.4mM K2HPO4, 3.3mM urea, 2.4mM NaH2PO4, and ascorbic acid (pH 6.8) (15).

-Surface hardness analysis

At the end of the first day of erosive cycles, final surface hardness (SHf) of the enamel blocks was measured as mentioned before. The %SHC was calculated as a percentage hardness change [(SHf – SHi)/SHi)] x 100.

-Profilometric analysis

At the end of the 5th day, enamel loss (µm) was quantitatively determined by a contact profilometer (Hommel Tester T1000, VS, Schwenningen, Germany), which presents accuracy around 0.5 µm. For profilometric measurement, the nail varnish was carefully removed using a scalpel and acetone solution (1:1 water) and the specimens were slightly dried. The diamond stylus was moved from the first reference to the exposed area and then to the other reference area (Lc=2.5 mm length). Four profile measurements were randomly performed in the center of each block. The vertical distance between the midpoints of regression lines on the reference and experimental areas was defined as erosive wear, and was determined using the software of the device (Hommel tester T 1000 - Turbo datawin-NT Version 1.34). The values were averaged (µm). The standard deviation of repeated analysis of a given block was 0.2 µm.

-Statistical Analysis

The assumptions of equality of variances and normal distribution of errors were checked for all the variables tested using the Bartlett and Kolmogorov–Smirnov tests (*p*=0.42 for %SHC and 0.25 for wear), respectively (GraphPad Prism software for Windows version 4.0, San Diego, CA, USA). The assumptions were satisfied and one-way Analysis of Variance was applied for both response variables (%SHC and wear), followed by Tukey’s test. The significance level was set at 5%.

## Results

Taking into account the initial erosion stage (1st day of erosive challenge) the data in  Table 1 shows that there were no significant differences among the groups for the response variable %SHC (*p*>0.05). Regardless of the cola modifications, all tested colas promoted similar enamel softening.

**Table 1:**
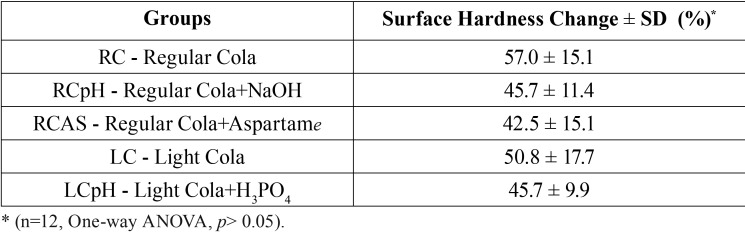
Mean and SD of the percentage of surface hardness change (%SHC) of the studied groups.

After 5 days of erosive challenge, enamel loss was detected in all groups (*p*<0.05) ( Table 2). Regular cola promoted significantly higher enamel loss when compared to light cola. The increase of pH of the regular cola resulted in similar behavior to light cola, thus this group showed significantly less enamel loss when compared to all other groups. On the other hand, the decrease of light colas pH revealed a significant increase in enamel loss, similarly to regular cola modified or not with aspartame, and higher wear compared to light cola and regular cola with increased pH. The addition of aspartame did not show a homogeneous reduction on enamel erosive wear, since the mean wear was significantly similar to light cola but also similar to regular cola and light cola with decreased pH.

**Table 2:**
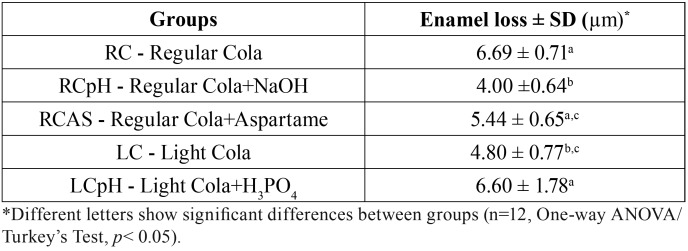
Mean and SD of enamel loss (µm) ± of the studied groups.

## Discussion

The terminology of “dental erosion” can be distinguished in “erosion” and “erosive tooth wear” to enable the differentiation between the two aspects of erosive process (16,17). Erosion represents the initial loss of structural integrity and mechanical strength caused by the effects of acid on the tooth surface, termed as enamel softening (16,17). The increasingly softened layer is prone to bulk tissue loss due to prolonged erosive challenge or the incidence of mechanical forces characterizing the erosive tooth wear (16,17). Thus, the present study evaluated the effects of cola drink modifications on initial (enamel softening) and prolonged (enamel wear) erosion.

The initial erosion lesion provoked using this *in vitro* model (cycling conditions, and a total erosive time of 8 minutes) showed softening of enamel surface and no tissue wear (the initial indentations were still present after the erosive challenges). Besides being a closed system without previous treatment with salivary pellicle, this model is in accordance to the guidelines for initial erosion models (18). The results from the softening analysis showed that there were no significant differences among the blocks surface subjected to the different cola drinks, modified or not. This result is in accordance with other study, which also did not find a significant difference between light and regular cola using an initial erosion model (7). However, an explanation for this result is not easy to be addressed, since difference on pH is a dominant factor in the prediction of erosive potential (8,19-22). In the present study, it was expected to find differences between the surface hardness changes among cola drinks with different pH. On the other hand, these differences were found for the enamel erosive wear. Thus, it seems that a severe erosive challenge is a more sensitive tool to screening possible factors affecting erosive potential under laboratorial conditions.

In addition to pH value, the literature identifies other chemical properties to be important in determining the erosive potential of a solution such as titratable acidity, buffering capacity, acid concentration, degree of saturation regarding apatite, calcium, phosphate and fluoride concentration, and inhibitors of erosion (8,23,24,25). A previous study showed that regular cola had a higher concentration of calcium and phosphate and similar buffering capacity when compared to light cola (13). However, the regular cola was more erosive than the light cola (12,13) as it was showed in the present data. Thus, the main differences between these colas, responsible for their distinct erosive potential, might be the pH values and the presence of sweetener (aspartame) and both of these hypotheses were tested in this study.

The results from the prolonged enamel erosive challenge showed that the increase of pH (RCpH) resulted in decrease of the erosive potential, reaching a similar behavior to the light cola (LC). Moreover, the decrease of the pH on light cola (LCpH) revealed a significant increase in the enamel loss, promoting similar wear when compared to the regular cola (RC). This finding is in agreement with available data that considers the pH a dominant factor in the erosive dissolution (8). Taking these aspects into account, it is possible to infer that the highest pH of the light cola is responsible for its less erosive potential. On the other hand, another study had demonstrated that non-cola drinks with higher pH values resulted in higher weight loss than cola drinks (26). It was also showed that regular cola and non-cola drinks resulted in higher weight loss than their diet version (26). Therefore, the erosive potential of the beverages could be dependent on not only the pH, but also the type of artificial sweetener present and amount of titratable acid. The pH value indicates the equilibrium measure of the hydrogen ion concentration; however, the overall acidic content of the drink cannot be evaluated by the pH (27,28). Besides the pH, the titratable acidity plays an important role in determining the erosive potential of soft drink, since it can estimate free hydrogen ions available to cause erosion (27,28). One limitation of the present study was that the titratable acidity of regular cola and light cola were not evaluated. However, a previous study that evaluated the erosive potential of soft drinks had observed that the soft drink Guarana, which presents worst chemical erosive characteristics of pH value (2.52) and titratable acidity (3.41 ml to reach pH 5.5, and 5.11 ml to reach pH 7.0), presented less enamel roughness than cola drink (pH 2.67 and tritable acidity of 0.56 ml to reach pH 5.5, and 1.87 ml to reach pH 7.0) (29). In that study, it was suggested that other substances present in guarana soft drink might have a protective effect in dental erosion (29). However, this result need to be analyzed with caution since the roughness is not the best method to evaluate enamel erosion and the most suitable methods for advanced erosion are surface profilometry and microradiography (30).

Previous studies had speculated that the less erosive potential by light cola in comparison with the regular one, could be due to the presence of inhibitors of erosion, which could be the sweetener aspartame (12,13). This hypothesis cannot be discarded since in the present study, the addition of aspartame to the regular cola slightly reduced its erosive potential. The regular cola modified with aspartame (RCAS) promoted enamel loss similar to the light cola (LC) but also similar to original regular cola (RC). Besides the significant difference between light and regular cola, the enamel loss values were not quite different as it was found in in situ studies (12,13). This limited antierosive potential of aspartame could be due to the *in vitro* model adopted that cannot completely represent the complex oral environment. We speculate that in an in situ condition the aspartame could modify the properties of the pellicle, maybe causing thickened layers, which could not be easily removed by acids. However, this hypothesis needs to be further clarified. In conclusion, the present *in vitro* study showed that the pH modification of cola drink has a major impact on its erosive potential during prolonged erosive challenges. However, the aspartame also has some influence on the erosive potential of the cola drink. Therefore, further in situ studies with enamel pellicle analysis should be conducted to better elucidate the role of aspartame on tooth erosion process.
